# Insights from 200+ years of personalized learning

**DOI:** 10.1038/s41539-018-0033-x

**Published:** 2018-09-05

**Authors:** David Dockterman

**Affiliations:** Harvard Graduate School of Education, 327 Longfellow Hall, 13 Appian Way, Cambridge, MA 02138 USA

## Abstract

Current initiatives to personalize learning in schools, while seen as a contemporary reform, actually continue a 200+ year struggle to provide scalable, mass, public education that also addresses the variable needs of individual learners. Indeed, some of the rhetoric and approaches reformers are touting today sound very familiar in this historical context. What, if anything, is different this time? In this paper I provide a brief overview of historical efforts to create a scaled system of education for all children that also acknowledged individual learner variability. Through this overview I seek patterns and insights to inform and guide contemporary efforts in personalized learning.

## In the beginning: competency-based schools

In 1779, Thomas Jefferson introduced a “Bill for the More General Diffusion of Knowledge” in the State of Virginia, calling for educational opportunities “without regard to wealth, birth or other accidental condition or circumstance.”^1^ Jefferson viewed public education as a critical safeguard “for the preservation of freedom and happiness.”^2^ Rising democracies needed literate citizens able to weigh options, make responsible choices, and protect themselves from tyranny. Jefferson’s bill failed, but it was an early part of an inexorable, but “uneven”,^3,4^ movement toward free, mass schooling that unfolded over the next five decades. Elwood Cubberley, in his 1919 history of education in the United States, notes the importance of the nation’s constitutional separation of church and state as critical to this movement: “Up to near the time of the outbreak of the War for Independence there had been but one real motive for maintaining schools—the religious.” Churches provided the bulk of any education for the poor, but the motives and obligations for schooling shifted with the rise of democracy.^4^ As the eighteenth century rolled into the nineteenth, the United States and other countries embraced a notion of public education, a state-sponsored commitment to educate all children, rich and poor.^5^ With this commitment to education for all, educators and policymakers found themselves needing a scalable, accountable mechanism for organizing masses of students and an affordable cadre of teachers to meet generally common educational objectives. How would they do it?

This state-sponsored commitment and mandate represented a departure from trade-based apprenticeships and private tutors. Prior to the American Revolution, access to education generally followed class lines. The well-to-do had personal tutors or attended private schools.^6^ The poor, particularly in New England, might receive rudimentary reading and writing instruction in church schools, but responsibility for education rested with the parents rather than the institutions.^7^ Colonists also brought an apprenticeship system with them from England,^8^ and poor children could well find themselves indentured apprentices. According to historian Merle Curti, “Cheap labor was scarce throughout the colonial period, and the apprenticeship system provided the better established classes with help at almost negligible cost,” while filling the demand for skilled labor.^6^ The democracy-fueled shift to educational enfranchisement, which gradually grew over the years to include women, people of color, and other previously marginalized populations, included a shift to government accountability. The demand and cost of teaching the masses could not be met with individual tutors and apprentice masters. Some other system of organization was necessary, a system that could be scaled quickly and held accountable for results.

John Lancaster, an English-born educational reformer at the turn of the nineteenth century who transplanted to the United States, developed a method to tackle this challenge. The Lancasterian, or Monitorial, System he created organized children by competence in different domains. Lancaster described his system in great detail in books he published to guide its uniform implementation from place to place.^9,10^ I provide a concise overview here.

Lancaster recognized that children came to school having acquired different knowledge and skills. Some may have been taught at home to read or do some math; others may have had little informal instruction. Once at school, some of these students learned quickly, while others progressed more slowly. To accommodate this variability, Lancaster broke the core disciplines of reading, writing, and math into instructional sequences and then sought to place each child in his appropriate place along each learning progression. A student, thus, might find himself at the entry level for writing, somewhere in the middle for math, and fairly advanced for reading. More competent students, called monitors, provided scripted lessons to develop skills and assessments to move each student to the next level in the sequence as soon as he demonstrated mastery. The lone teacher managed the process, focused on the children at the beginning of the learning sequences, and dealt with issues as they arose.^9^ Today we might call what Lancaster devised 200 years ago a self-paced, mastery-based, peer-learning instructional system. That description sounds tantalizingly close to some of the visions espoused by current personalized learning advocates. The U.S. Department of Education, for instance, defined competency-based learning or personalized learning as a system that “allows students to progress as they demonstrate mastery of academic content, regardless of time….”^11^

Organizing students by competence and distributing instruction across the student population helped address the cost of teachers^4^ and the anticipated variability in student knowledge and skills. A sequenced set of preset lessons supported rapid scalability, with untrained monitors able to deliver them. The system spread across the United States and other nations in the early nineteenth century. (In his 1821 update of the monitorial system, Lancaster summarized the implementation of his model in Ireland, France, Russia, Spain, Germany, Asia, Africa, South America, and the West Indies, as well as England and the United States.^10^) In 1894, C.S. Boykin, of the U.S. Bureau of Education, recalled that in the early 1800s “instruction was almost wholly individual.” When a student entered a school, “no matter at what time of the year,” he would simply pick up where he left off with his last lesson. “If he had been through Webster’s ‘Blue-back’ Speller twice, and had finished the last column of the tenth page, on the third round, the first column on the eleventh page would naturally be the first lesson that his new teacher would give him,” joining students who were working on the same lesson.^12^ The system, thus, could be held accountable for producing students who met mastery objectives.

Grouping children by what content they had completed, though, neglected other differences that impacted their learning. Lancaster, for example, anticipated that his system could undermine motivation for older, less capable children who found themselves grouped with younger, more capable ones. To address the potential social stigma, Lancaster suggested sometimes placing children with their similarly aged peers, even if the placement was beyond their ability, “to prevent depression and discouragement to senior boys.”^10^ Lancaster also anticipated that engagement in learning might wane due to other social pressures, as in the case of “A boy, named Harvey, [who] was once allured into bad company, in whose society, his lively, playful mind found more pleasure than in school.”^10^ The reformer described a father’s forceful response to Harvey’s nonacademic viewpoint along with a teacher’s more kind and incentive-laden reaction. Neither approach had any lasting impact. Lancaster went on to suggest ways to leverage group competitions, including a wager between student and teacher, to draw Harvey back into the learning community. The monitorial model grouped students according to common content needs while anticipating and planning responses for other psychological and social factors that could affect learning.

A promising educational technology tool that was introduced into this dominant instructional model offers some valuable insight for today’s Ed Tech advocates. In the early decades of the nineteenth century, educators in the United States began describing an incredible new tool for engaging instruction: the blackboard. Observers at the time effused about the illustrative power of chalk in the hands of an animated instructor. After watching Professor Claude Crozet, a French officer, teaching geometry with a blackboard at the Academy at West Point in 1817, one viewer wrote: “we know of no mere adjunct of teacher, so useful as the blackboard.”^13^ While books are passive, the blackboard/teacher combination provided a mechanism for the dynamic unfolding of a lesson in whole-class instruction. Reformers pushed for blackboards to be included in the common schools that were being constructed to meet the needs of this bold commitment to public education.^14^ The results, though, disappointed the enthusiasts. An 1839 report on chalkboard use concluded that “Blackboards are not uncommon, but are but little resorted to by the teacher.”^15^ Another report in 1842 complained that the teacher “knows almost as little how to use it as his pupils.”^16^ From today’s perspective it’s a bit baffling to imagine teachers not knowing how to use a chalkboard. The charges against these nineteenth-century teachers sound similar to those against late twentieth- and early twenty-first-century teachers regarding digital technologies.^14,17^ Were chalkboards as technically baffling as computers?

These early examples of chalkboard use came from places like West Point Academy (a 4-year college founded in 1802 to train military leaders) and other institutions of advanced learning. In these settings, a single teacher instructed large groups of students in the same content. In monitorial schools, however, teachers, and their monitors, worked with small, competency-based groupings of students. Lancaster provided explicit directions on the use of “small slates” by individual students,^9^ but his guides never mention a chalkboard. Teachers rarely, if ever, taught everyone at once. Instructional technology designed for whole-class instruction made no sense. The blackboard didn’t fit the system, and the system, and the teachers in it, rejected it.^14^

### Dominant pedagogy matters

As my historical journey will illuminate, the organization of schooling and pedagogy did change in a way that made chalkboards an indispensable instructional tool. However, until that shift, whole-class technology was a mismatch for the monitorial system. Similarly, digital technology for individualized or targeted small group learning may be a mismatch for a system built around teaching same-aged students the same content at the same pace. Historian Larry Cuban’s analysis of educational technologies from 1920 to the 1980s exposed a tendency for teachers to subvert new technology associated with new methods of teaching to support their existing instructional patterns.^17^ Systemic-based resistance may be inevitable when the dominant model of instruction isn’t aligned with the desired reform. Pedagogical shifts, though, are possible.

## Shifting structure, shifting pedagogy

The monitorial model proliferated in rural settings with small numbers of students that a single teacher could know, organize, and support. In urban settings, though, Lancaster’s system became difficult to manage. Because Lancasterian schools recognized multiple levels of knowledge variability, they tended to have lots of groups. A nineteenth-century commentator noted that “a teacher with a school of moderate size, containing pupils of all ages, sexes, and sizes, might easily have fifty or sixty [groups].”^18^ Dealing with so many groups became particularly unwieldy in urban schools with lots of students. Educational historians of the late 1800s and early 1900s describe an almost natural progression in these growing cities from a one-room schoolhouse bursting with clusters of students to a seemingly more manageable grouping system.^4,18^

Expanding populations of school-aged children required more spaces and more teachers, leading to larger common groupings and a level of teacher specialization.^18^ Age-grading, according to William Shearer, a late nineteenth-century education reformer, dates back to 1537 in Europe.^18^ The system evolved in the United States over the second half of the nineteenth century in response to this population growth and eventually became the norm for educational institutions there and around the world. The practice of bundling together students with similar birth years offered the apparent efficiency and effectiveness of professional teachers trained to deliver age-appropriate content, compared to peer monitors who were students first and teacher surrogates second.^18,19^ Similarly aged children were generally expected to have the same knowledge and progress at the same pace, so they could be taught at once as a group. A change in pedagogy followed the change in school organization. Within this context, the chalkboard did become an “indispensable” tool of instruction.^14^ (It wasn’t perfect, though.) Teachers did have to turn their backs on their students to write on the chalkboard, which sometimes led to discipline issues. A key promoted feature of the overhead projector was that it could be used while the teacher still faced the students, maintaining order.^20^

Unfortunately, similarly aged students didn’t progress as uniformly as reformers had hoped. Cubberley wrote in 1916 that 70.4% of students should be progressing according to graded level expectations. About half the remaining students would be lagging (called “retardation” in those days) and half would be accelerating.^21^ The system was designed around the 70%, a goal that often was not met.^22^ According to Tyack and Tobin, this “batch processing” of students “created a category of organizational deviant: the ‘retarded’ or slow student who failed of promotion.”^19^ These issues were apparent early on. Shearer wrote in 1898: “…by far the largest number of schools are supposed to be graded for the ‘average pupil.’ At first sight this looks reasonable: but, could anything be more absurd?” He goes on to describe a range of reforms to accommodate variations in pace of learning, including allowing students to switch into faster or slower moving classes, ability tracking, and flexible student reclassification.^18^ Attempts to respond to individual student difference continue today with remediation courses, gifted programs, and, more recently, response to intervention. Still, many students remain exceptions to the expected norm.

That so many students failed to progress with their peers led to a repeat of the social stigma issue that worried Lancaster. Cubberly, like Lancaster, feared that failure “tends to destroy self-confidence,” leading to a negative spiraling effect. In a competency-based system, moving slowly in one area doesn’t hold the student back from advancing in another. In an age-graded system, on the other hand, it’s all or nothing. The student either moves on to fourth grade or repeats a year in third grade. The stigma of failing a grade is theoretically higher than the potential embarrassment of moving slowly through one of several competency progressions. Schools felt pressure to socially promote students or maintain parallel tracks with different standards. The results are very familiar. Critics of overpromotion in Philadelphia in 1948 complained “that some pupils who should still be in grade school are in high school and many high-school pupils graduate without the fundamentals.”^23^

### Scalability requires systems for management and accountability

Public education doesn’t teach one child; it teaches all children, including children who may be unmotivated about school, children with learning issues, children from impoverished environments, children who don’t speak the language of instruction, and so on. Responding to those various needs in small settings is challenging but much more manageable than meeting those needs in densely populated situations. I would argue that sorting students by age was not an unreasonable organizing principle for addressing this unwieldy management situation at the time. It allowed professionally trained individuals to teach common content to children who theoretically shared similar developmental needs. It also offered a relatively straightforward way to track the success of the system: how many students moved from one grade to the next each year? Unfortunately, the age-graded system has never really worked that well for a substantial number of children.

## The promise of individualized instruction

Nonetheless, age-graded schools became the overriding structure for public schools. Accountability systems, organized around grade-level expectations, tracked student progress from grade to grade. Retention and graduation rates, along with standardized tests, became common metrics for measuring school success.^24^ The system was constructed around the assumption that most students would need the same instruction and acquire the same content at about the same time. Within this system, flickers of the earlier competency-based approach lingered.

In the 1950s, ‘60s, and ‘70s, non-age-graded elementary schools attempted a comeback.^25^ Advocates of these renewed nongraded schools didn’t promote their reforms with nostalgic visions of the one-room school house. Instead, the movement often attached itself to a push for individualized and more learner-centered instruction, incorporating “the extensive use of learning stations, learning activity packets, and other individualized, student-directed activities.”^26^ Those revised nongraded schools look even more like today’s renditions of personalized learning, working to match instruction to variable student content and skill needs while also embracing increased learner agency and motivation.

Two other “innovations” during those decades shared a number of goals with this nongraded renewal effort and may have collectively contributed to what is emerging today. One of those innovations is captured in a 1960s advertisement from Republic Steel touting a bold new vision for education. “Individualized instruction, the ultimate dream of effective education” was just around the corner. A single computer, in this case a massive mainframe housed in an air-conditioned room made of steel (hence the enthusiasm of Republic Steel), would deliver “individual instruction to scores of students—in a dozen subjects at the same time.”^27^ The promise that computers could provide the right content to the right student at the right time has been around for over half a century. Realizing that promise has been elusive, but it continues to entice many by offering a potential solution to the management dilemma faced by any attempt to meet the personal needs of a large group of learners. Computers can be the tutors, the smart monitors, edging individual students forward at their own pace. The teacher would be free to manage the process and respond to the other factors (such as the issues of engagement and humiliation avoidance that have dogged the monitorial and graded school systems) that influence learning. That combination sounded good in the 1960s; it still sounds good as we roll into the 2020s.

Those other factors impacting student learning, though, can be many, varied, and very important. A second innovation of those decades involved defining the multitude of characteristics that can impact a student’s ability to learn. In the 1970s, Cronbach and Snow launched an approach called Aptitude Treatment Interaction (ATI) to provide rigor to research on the intersection of learner and instruction.^28^ ATI recognizes that learners can and do differ in lots of ways—cognitively, psychologically, and environmentally. It’s not surprising then that individuals will respond to different tasks and different forms of instruction in different ways. What engages and informs one student might provoke crippling anxiety in another. Indeed, the same learner might respond to the same intervention differently under different environmental or psychological conditions. ATI described methodological techniques for determining the most effective match for each student, but the combinations of potential variables and treatments were, according to Cronbach and Snow, “virtually inexhaustible”.^28^

Cronbach and Snow’s work laid the foundation for some of the later research and development in the world of computer-based, adaptive learning.^29^ The ATI framework helped organize digital efforts to direct the right treatment matched to a particular student need under the optimal conditions. ATI’s expansive view of relevant learner aptitudes, beyond acquired knowledge and skill, has helped expose the psychological needs of students, long recognized by educators, as central to instructional considerations. It isn’t enough to scale an instructional system around a single aspect of learner need, like content competence or social acceptance. A robust personalized learning model must respond to whatever needs matter for each individual learner (The Collaborative for Academic, Social, and Emotional Learning, https://casel.org).^30,31^

Responding to the vast range of potential learner needs, though, is really difficult to scale, particularly in a system designed around the assumption that most students would learn the same content in the same way at the same pace. A review of the efficacy of the 1960s and ‘70s’ nongraded schools did show improved student performance.^26^ The movement, though, sputtered. Historically, individual schools have had success with instructional models that accommodate multiple dimensions of student variability, but a scalable system never emerged. The competency-based Lancasterian model of the early nineteenth century was difficult to manage with large student populations. In addition, the shift to age-graded schools included a shift in dominant pedagogy built around treating clusters of students uniformly. Are we stuck?

## Twenty-first century personalized learning: guided by historical insights

“Personalized learning is hard.”^32^ So begins a 2017 news summary of research^33,34^ on contemporary efforts to create schools that incorporate mastery-based learning along with a commitment to meet individual student needs and interests. Given the history described above, maybe the more appropriate quote is, “Personalized learning is *still* hard.” The technology promising to support individualization is much more robust and available now than it was in the 1960s and ‘70s, let alone the 1820s. Funding and enthusiasm from the Gates Foundation, the Carnegie Foundation, the Chan−Zuckerberg Foundation, and others is plentiful. In 2016, for instance, Education Week estimated that the “U.S. Department of Education has given half a billion dollars to districts that embrace” personalized learning and that “since 2009, the Bill & Melinda Gates Foundation has committed $300 million to support research and development around personalized learning”.^35^ In addition, Universal Design for Learning (CAST, http://udlguidelines.cast.org/) provides an accepted framework for organizing and addressing multiple dimensions of learner variability. The growth- and learning-mindset academic communities have aggregated and disseminated research related to how sense of purpose, growth mindset, and belonging influence student academic performance and ability to learn (The Mindset Scholars Network, http://mindsetscholarsnetwork.org/research-library/). Advocates for Social Emotional Learning (The Collaborative for Academic, Social, and Emotional Learning (CASEL), https://casel.org/what-is-sel/ among others) seeks to define a set of competencies that support the learning process. These efforts, in combination with ongoing research in The Learning Sciences,^36^ have deepened understanding and awareness of multiple dimensions of learner variability that impact motivation, behavior, and academic performance. All these advances in tools and knowledge, and we’re still living with small pockets of often modest success while clinging to the hope of a major revolution. Can we leverage any insights from the past two centuries to guide and inform current efforts? I offer some suggestions below.

### We need to foster a new dominant pedagogy

The instructional model of our age-graded system is based on the assumption of *sameness with exceptions*. Publishers of instructional materials and teachers construct lessons for the group and differentiate to accommodate the outliers. They provide layers of typically predetermined intervention to capture lagging students and occasionally offer extra challenges or gifted programs to stave off the boredom of children who are ready to progress ahead of their peers. The core of teaching—whether for the theoretical 70% of average students, the 15% who are “retarded”, or the 15% who are accelerated—is focused on the age group organized into classrooms.

A personalization-based pedagogy, on the other hand, starts with the assumption that each student is different. Variability, across multiple dimensions (not just domain knowledge and skill), is inevitable. Allowing older, lower-performing students to remain with their peers, whether through Lancaster’s reassignment or age-graded social promotion, reflects an historical awareness of the role of affect on student learning. Today, though, we know much more about the role of mindset and emotion in the learning process.^37^ We continue to unravel the importance of executive function and the self-regulation of learning behaviors.^38^ Research has also informed us about which self-regulated learning strategies are effective.^39^ And ongoing research should not simply look for instructional interventions that have the broadest impact on student populations; it should identify under what conditions each intervention works with different learner characteristics. It’s about matching an instructional action with the right learner at the right time. Instructional design and materials, informed by data and learning science, can focus on the anticipated variability within the target population that will matter *most* for learning and demonstrating competence with the academic goals. Instead of waiting to see who doesn’t succeed in the group lesson, learning environments and related tasks, often supported by technology, can be constructed to remove expected barriers and bolster supports to address anticipated needs, including affective and behavioral ones, and both proactively and reactively. Teaching and learning in this system won’t be uniform: one would never expect all teachers to teach the same lesson on the same day in the same way to all students.

### To sustain this new approach, a new organizational and accountability system must mesh with the new pedagogy

As I’ve noted above, an educational infrastructure that expects the same academic progress for children of the same age reinforces a pedagogy that generally treats and instructs similarly aged children the same way from school to school, state to state, and even across nations. A professional class of teachers is certified based on grade-level specialties. Assessments measuring success at meeting age/grade-level expectations readily facilitate comparisons and provide indicators of how well the system is being implemented. The system is efficient and scalable, even if it isn’t uniformly effective. A pedagogy based on individual learner variability may be able to exist here or there as an exception to the system, but, given past attempts, it is unlikely to scale.

What kind of scalable educational structure *can* support and encourage a pedagogy more directed toward meeting variable learner needs? Contemporary experiments abound. The State of New Hampshire and a number of individual school districts have begun moving toward reporting on mastery of individual learning objectives rather than providing a single grade encompassing all grade-level expectations or all domain-specific grade-level expectations.^40^ Summit Public Schools, a collection of high schools targeting a personalized learning approach that addresses content as well as learning skill and behavior objectives, offers a structure that attempts to respond to students’ academic and emotional needs in a mastery-based system.^41^ MAPLE, a Massachusetts consortium of personalized learning schools, supports individual school efforts to shift local systems within the existing state framework (MAPLE, http://learnlaunch.org/MAPLE/). The League of Innovative Schools, under the umbrella of the nonprofit organization Digital Promise, is exploring alternative models (Digital Promise, https://digitalpromise.org/initiative/league-of-innovative-schools/). All of these efforts include shifts in accountability, goals, instructional materials, pedagogy, and sometimes space. They are comprehensive at varying levels—from school, to district, to state. Will an acceptable, scalable model, along with a matching accountability infrastructure, emerge from this swirl of experimentation?

### It won’t be easy

Creating a mass system that accommodates individual variability successfully for each learner has confounded public education for over two centuries. It may be impossible to organize around the “virtually inexhaustible” combinations of variation and treatment involved in educating each child en masse. The competency-based organizing principle of the Monitorial System, even with its rigid and rote lessons, turned out to be too much of an organizational burden for urban schools. The age-graded system adopted in response has struggled to meet the needs of even a 70% target of the student population. The search for the appropriate grouping commonalities among a sea of differences continues. Maybe we should think in clusters of ages—primary, elementary, middle, and high. We expect primary students to move into elementary in *about* 3 years. Some may move more quickly and some more slowly. Maybe the whole system is individualized with much of the instructional and tracking responsibility turned over to computers. Students “finish” when they finish.

We need to try different approaches, and in light of the historical insight that the task is difficult, we need to give the transition time. A change in infrastructure and pedagogy doesn’t happen overnight. Even before we had an entrenched educational organization, the shift from a one-room schoolhouse methodology to an age-graded one took decades. We have much to do and undo. Fortunately, just as bulging urban populations and a developing manufacturing economy strengthened the attractiveness of an age-graded school organization in the nineteenth century, current demographic and economic shifts may help accelerate organizational change in today’s schools. A 2013 study at Oxford University estimated that 47% of jobs in the United States were at risk of automation.^42^ A special report in *The Economist* in 2017 predicted employment turmoil for both skilled and unskilled workers as technology and robotics invade the workplace.^43^ Disruption in the workforce may lead to sustained disruption in the institutions feeding labor into that workforce. Current experiments with competency-based education may have more than the desire for equity and pedagogical reform pushing it forward.

Whatever the forces at work, complicated change is difficult. Patience, perseverance, and resilience are all essential. Public education has always left children behind. Applying insights from the past in combination with emerging knowledge from the learning sciences and the promise of digital technologies should enable us to carry more of those children forward.
